# The Effect of Growth Conditions on the Seed Size/Number Trade-Off

**DOI:** 10.1371/journal.pone.0006917

**Published:** 2009-09-10

**Authors:** Cloé Paul-Victor, Lindsay A. Turnbull

**Affiliations:** Institute of Environmental Sciences, University of Zurich, Zurich, Switzerland; CNRS UMR 8079/Université Paris-Sud, France

## Abstract

**Background:**

If the amount of resources allocated to reproduction (*K*) is fixed, then an increase in seed mass (*S*) can only be achieved by a decrease in seed number (*n* = *K/S*). Thus, log(*n*) = log(*K*)−log(*S*) producing a slope of −1 when seed mass and number are plotted on log-log axes. However, in comparative studies, empirical support for a slope of −1 is limited and contentious, leading some to question the utility of this concept.

**Methodology/Principal Findings:**

First, we show that the expected slope depends on whether genotypes and species producing seeds of different mass are expected to reach the same adult size and that this in turn depends partly on the nature of growth. Second, we present experimental results using a population of recombinant inbred lines (RILs) of *Arabidopsis thaliana*. When these RILs are grown in large pots with plentiful nutrients, they exhibit a trade-off between seed size and number with a slope of −1.68 (±0.18) on log-log axes. This occurs because of genetic correlations between seed mass and adult size so that, under the right growth conditions, lines producing lighter seeds have the genetic potential to produce larger rosettes and hence a greater total mass of seeds. We re-grew lines in small pots (10 and 40 mm diameter) in a nutrient-poor substrate so that final adult size was heavily restricted by pot size.

**Conclusions/Significance:**

Under our growth conditions, small-seeded lines were unable to produce a greater total mass of seeds. Hence a trade-off emerged between seed mass and seed number with a slope of −1.166±0.319 on log-log axes in 40-mm diameter pots (close to the expected value of −1), although the slope was 0.132±0.263 in 10-mm diameter pots, demonstrating that the nature of the trade-off is sensitive to the growth conditions.

## Introduction

Smith and Fretwell [1] first argued that a trade-off must emerge between the number and size of offspring if individuals have a fixed amount of resources to allocate to reproduction. Thus, if an adult plant has *K* resources to allocate to reproduction, the number of seeds produced (*n*) is given by:
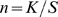
(1)where *S* is the seed mass. Equation 1 produces the seed size/number trade-off because any increase in *S* must result in a decrease in *n*. While equation 1 is undeniably true for a given individual, equation 1 also predicts that such a trade-off might occur among individuals producing seeds of different sizes, including individuals belonging to different species. Thus, if the number and size of seeds is recorded from a range of individuals and plotted on log-log axes, we expect a slope of −1:
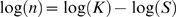
(2)


However, empirical evidence for such a trade-off in plants is limited and contentious [2], [3], [4] leading some to question the utility of this concept [3], [5] and see [6].

One reason for the observed deviations from the expected trade-off is that comparisons are often made among species from different communities with different life-forms or productivities [5], [7], [8]. We show here, however, that even when comparisons are made among annual plants growing under similar conditions, the final mass available for reproduction (*K*) depends on the nature of growth and competition, and that the trade-off may therefore still be masked. We then report the results of a controlled experiment using inbred lines of *Arabidopsis thaliana* that differ in their seed sizes and demonstrate the dependence of the trade-off on the growth conditions.

### Case 1: constant adult size

For the trade-off to be satisfied, the final size of individuals must be roughly constant and independent of seed size. This is most likely to occur when plants are grown in pots without further nutrient addition, where plants often follow a logistic curve [9]. In this case, final size is probably strongly constrained by the pot and may be largely independent of initial (i.e. seed) mass–for example, Susko and Cavers [10] showed that individuals from large seeds were still larger after 15 days of growth in pots but that such differences disappeared at later dates. Similarly, final size may be independent of other growth characteristics (e.g. growth rates, photosynthetic rates, maximum rosette size etc.).

### Case 2: exponential growth

At the other extreme we could assume that, rather than being space-restricted, plants grow for a fixed time interval, either because plants follow age-dependant rules [11], [12], [13], [14] or because the environment only allows a limited number of days for growth. For example, as a crude (and undoubtedly untrue) approximation, let us assume that isolated plants can achieve exponential growth in the absence of competition. Exponential growth has apparently been observed for isolated plants of desert annuals growing in their natural environment [15] while Turnbull et al. [16] found that sand-dune annuals grown in pots had a short initial phase of exponential growth. If plants grow exponentially, then they achieve a mass at time *t*, 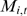 given by:



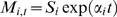
(3)where 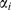 is the intrinsic growth rate. Notice that, in contrast to the pot-grown plant, such growth is always mass limited and hence carbon limited (a bigger plant can always achieve a higher growth rate, in contrast to the plant following the logistic curve). If we assume that the growth period, *t*, is the same for all species or genotypes, and that all individuals have the same intrinsic growth rate, 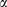, then if two species *i* and *j* begin from different seed mass, the ratio of their final masses is given by:
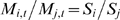
(4)


Thus, if genotype *i* has twice the seed mass of genotype *j*, it will also have twice the final mass and can produce the same number of seeds:
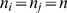
(5)as 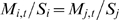 (from eqn 4) and the trade-off between seed size and seed number seems to have disappeared. However, as the area of ground that must be exploited to achieve a given final mass increases in direct proportion to the final mass of the plant, the trade-off would in fact appear per unit area of ground, rather than per individual [17], [18]; thus, small-seeded genotypes would produce more seeds per unit area instead of per plant. While the approximation of exponential growth is no doubt imperfect, perfectly size-symmetric competition [19]–in which the size hierarchy does not change throughout the growth period [19], [20], [21]–would also preserve any initial size hierarchy due to seed size differences. Thus, without knowing more about the nature of growth in a particular context or environment, it is difficult to know at which level the trade-off should be searched for.

### Finding the elusive trade-off

If pot-grown plants are space restricted and achieve similar final size, then a slope of −1 (eqn 2) should at least emerge among individuals growing in pots; however, in reality, plants are often grown in large pots with regular nutrient addition. Under these conditions it seems reasonable to suppose that final size might differ among individuals with different seed sizes or growth characteristics. For example, the model plant *Arabidopsis thaliana* is a strict annual and at some point the plant flowers, makes seeds and dies. By delaying flowering plants can continue to make larger rosettes, and if they are not nutrient-limited, this additional leaf area will probably lead to further increases in growth rate by increasing carbon capture. Thus, if nutrients are plentiful, the final reproductive mass (*K*) might be related to those characteristics which allow plants to build larger rosettes, such as delayed flowering time [22] and longer maximum leaf length. For example, in a population of Recombinant Inbred Lines (RILs) derived from crosses between two parental lines differing greatly in their seed size [23], [24], small-seeded lines were found to flower later, build larger rosettes and produce a greater total mass of seeds when grown in large pots with plentiful nutrients. This led to a negative relationship between seed mass and the total mass of seeds (Slope = −0.69±0.184); hence, the slope of the relationship between seed size and number on log-log axes was considerably steeper than −1 (−1.68±0.18; *F_1,160_* = 84.4, *p*<0.0001; calculated from data in [23]). This relationship probably occurs because of genetic correlations between seed size and vegetative traits within the RIL population [23].

To test whether the trade-off could be manipulated by changing the growth conditions, we grew individuals from the same RIL population in small pots on a poor sand substrate without further nutrient addition. By restricting growth, we hoped to make adult size, and hence total reproductive mass, a function of pot size only, and hence break the link between adult size and seed size. If successful, we expect to see a trade-off between seed size and number with a slope of −1 on log-log axes.

## Materials and Methods

### Plant material

We exploited natural genetic variation in the model plant *Arabidopsis thaliana* [25], [26], [27], [28]. We selected a set of RILs derived from reciprocal crosses between two parental lines: Landsberg *erecta* (L*er*), obtained as a mutant (*er*) from an accession of northern Europe [29], [30], and Cvi, an accession from the tropical Cape Verde Islands [31]. The two parents L*er* and Cvi have, respectively, small and large seeds (L*er*: 1.93 mg±0.10; Cvi: 3.51 mg±0.08; mass per 100 seeds, mean±1 SD; [23]. The range in mean seed mass exhibited by the original lines described in Alonso-Blanco et al. [23] is 1.45–3.73 mg/100 seeds and is greater than the variation expressed by the two parents. We selected 30 RILs from the possible set of 162, plus the two parent lines, for the experiment described here. The 30 lines were selected by dividing the original 162 lines into six equally-spaced seed mass groups and selecting five lines at random from each group. Half of the selected lines carry the *erecta* mutation inherited from the L*er* parent, while the other half carries the wild-type *ERECTA* allele (Table S1). The most striking feature of lines carrying the *erecta* mutation is their reduced height (phenotype curated by the Arabidopsis Biological Resource Centre (ABRC)).

### Experimental design

**Figure 1:**
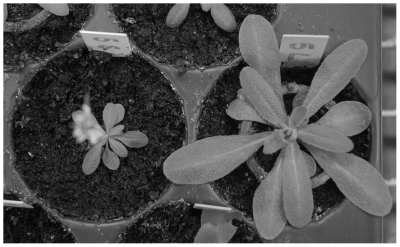
Plants grown in 10 and 40 mm diameter cylinders inserted into cells within a single flat. The two plants shown are genetically identical (from the same line). Note that the surface area available to growing rosettes is exactly the same for both treatments.

The seeds were obtained from The Arabidopsis Information Resource (TAIR) and we weighed a single sample of 100 seeds from each of the 32 selected lines (range: 1.286–4.107 mg/100 seeds). This is referred to as *sown seed mass*. To provide different degrees of belowground growth restriction, all lines were grown in both small (10 mm diameter) and large cylinders (40 mm diameter) inserted into standardised cells (65 mm diameter) within a flat completely filled with compost. Each flat contained 35 cells and was 70 mm deep. The cylinders allowed us to randomise pot diameter treatments within flats and ensured that the spacing of individuals in different pot sizes and the surface area available to growing rosettes was exactly the same (Figure 1). Rosettes from neighbouring cells were never observed to overlap. We aimed to have five replicates of each line and pot size combination in a blocked design but due to germination failures the final design was slightly unbalanced.

Pots were sown with four seeds and thinned as soon as seedlings emerged to leave one plant per pot (the most central healthy seedling). The plants were grown in a glasshouse with both natural light and additional artificial lighting when the natural light was below 25 kLux and kept under a cycle of 16 h light (22°C) and 8 h dark (20°C). When the plants began to produce fruits, we put perforated bags around the inflorescence to collect all the seeds produced by each plant. We continued watering until we observed complete senescence of all plant parts: 78 days in total. After 78 days, all seeds from each plant were weighed to give the *total mass of seeds*. In order to estimate the total number of seeds produced by each plant (*harvested seed number*), we divided the total mass of seeds per plant by the estimated seed mass of each plant. The seed mass of each plant (*harvested seed mass*) was calculated by weighing a single sample of 100 seeds collected from each individual (determined to the nearest microgram). Seed masses are presented throughout the paper as the mass of 100 seeds.

### Statistical analysis

We analyzed harvested seed mass, harvested seed number and the total mass of seeds in relation to both sown seed mass and harvested seed mass. By fitting sown seed mass as the explanatory variable we can assess the fitness consequences of starting life from a particular seed mass, while analyses using harvested seed mass as the explanatory variable assess the fitness consequences of *producing* seeds of a particular mass. To facilitate comparison with Alonso-Blanco [23] and because sown seed mass was only available as a mean per line, harvested seed mass was also calculated and fitted per line when used as an explanatory variable; however, the response variables were always calculated and analysed per-individual. All analyses were carried out using linear mixed-effects models in the statistical package R using the *lmer* function [32] in which we followed the model-building approach outlined in Pinheiro and Bates [33]. For the fixed effects we first assessed the approximate significance of terms using F-tests from a linear model with the appropriate error terms. The final significance was assessed using t-tests from the table of coefficients in a mixed-effects model which only retained these significant terms (although the two approaches never disagreed). In the mixed-effects model, line and block and their interactions with other terms were fitted as random effects. The significance of the random effects was judged using likelihood ratio tests and non-significant terms were removed. The variables harvested seed mass, harvested seed number, total mass of seeds and sown seed mass were all log-transformed to meet the assumptions of the analysis and because expected relationships are on log-log axes (see Introduction). However, means and differences between means are presented on the original scale. Differences between means are presented with their 95% confidence interval (CI).

## Results

### Overview

**Figure 2:**
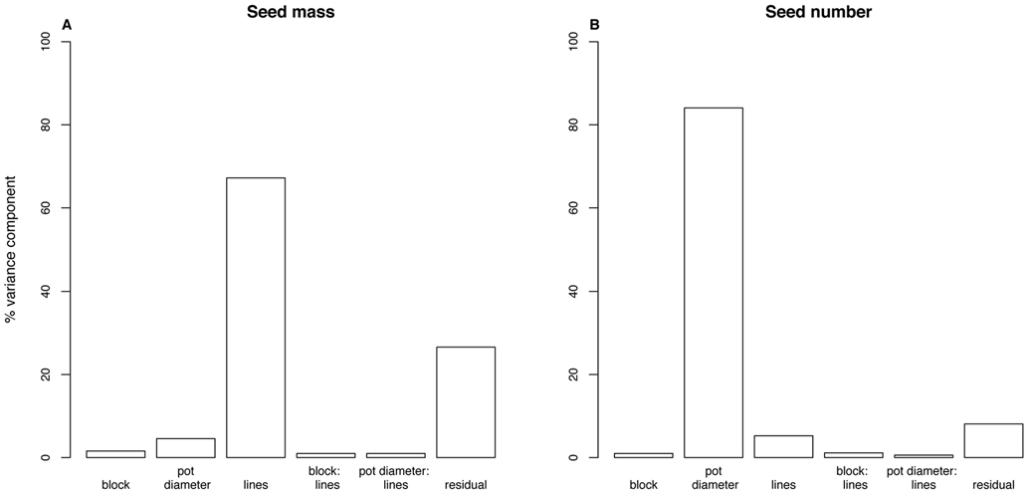
Results of a variance components analysis of harvested seed mass (A) and seed number (B). Variance components are expressed as percentages of the total in each case. Note that seed mass shows a large genetic component (variation among lines) whereas seed number shows a large environmental component (variation among pot sizes).

We begin by fitting a model for both harvested seed size and harvested seed number with all terms fitted as random effects, as recommended by Gelman and Hill [34]. This provides a general overview of how the variance is partitioned between the various possible terms and their interactions. As expected, most of the variance (67%) in harvested seed mass exhibited by individual plants is due to lines: i.e. seed mass is under strong genetic control (Figure 2A) which explains the highly significant correlations obtained between our data and previous datasets (Table 1). In contrast, most of the variance in harvested seed number (85%) is due to pot diameter, i.e. to the environment (Figure 2B). Interestingly, the correlation between seed number in our experiment and a previous dataset are weaker (Table 2), indicating that lines that performed well in our experiment did not necessarily perform well in a previous experiment. The interaction between the genetic and environmental component appears to be very small in both cases (<1%, Figure 2).

**Table 1:** Correlations (all significant at *P*<0.0001, *n* = 30) between the mean seed mass recorded for each line in a previously published study (Alonso-Blanco et al., 1999), seeds obtained from the Arabidopsis centre TAIR (sown seed mass) and seeds produced in the experiment reported here (harvested seed mass) in two pot sizes.

*Variables*	Published seed mass	Sown seed mass	Harvested seed mass (10 mm diameter pots)	Harvested seed mass (40 mm diameter pots)
Published seed mass	1	0.885	0.850	0.770
Sown seed mass		1	0.778	0.756
Harvested seed mass (10 mm diameter pots)			1	0.870
Harvested seed mass (40 mm diameter pots)				1

**Table 2:** Correlations (* *P*<0.05, ^NS^ non significant, *n* = 30) between the mean seed number recorded for each line in a previously published study (Alonso-Blanco et al., 1999) and the mean seed number produced by plants in the experiment reported here (harvested seed number) in two pot sizes.

*Variables*	Published seed number	Harvested seed number (10 mm diameter pots)	Harvested seed number (40 mm diameter pots)
Published seed number	1	0.133 ^NS^	0.491 *
Harvested seed number (10 mm diameter pots)		1	0.396 *
Harvested seed number (40 mm diameter pots)			1

### Detailed analyses

**Figure 3:**
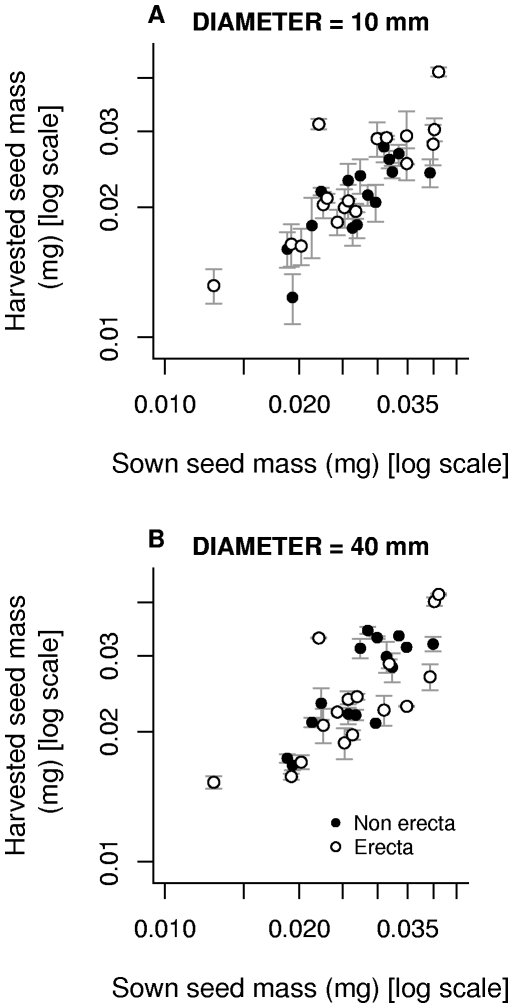
The relationship between sown seed mass and harvested seed mass in both pot sizes. Points show the mean (±1 s.e.m) of all individuals belonging to the same line.

The relationship between sown seed mass and harvested seed mass was strongly positive (Figures 3A–B) for both pot sizes with a common slope of 0.81 (±0.217). There was an interaction between the *erecta* mutation and pot size (F_1,28_ = 5.72, *p* = 0.0237, Table 3): wild-type *ERECTA* lines produced seeds that were on average 0.155 mg (CI: 0.0836–0.231 mg) heavier in 40-mm than in 10-mm pots, a difference of around 16%; in contrast *erecta* lines produced seeds that were on average only 0.0517 mg (CI: 0.00909–0.116 mg) heavier in 40-mm than in 10-mm pots; a difference of around 3.3%. Thus, lines carrying the *erecta* mutation appear to have reduced phenotypic plasticity in seed size. For the random effects, the pot size × lines interaction was effectively zero, but variation among lines was again large (χ^2^ = 65.9).

**Table 3:** ANOVA of harvested seed mass with sown seed mass fitted as an explanatory variable.

Term	Error term	Df	Sum Squares	Mean Square	F	P
Pot diameter	R	1	0.305	0.305	12.2	<0.0001
*erecta* mutation	L	1	0.00210	0.00210	0.0125	0.912
log (Sown seed mass)	L	1	11.1	11.1	66.3	<0.0001
Lines (L)	R	29	4.87	0.168	6.73	<0.0001
Pot diameter : *erecta* mutation	P:L	1	0.124	0.124	5.72	0.0237
Pot diameter : log (Sown seed mass )	P:L	1	0.00580	0.00580	0.268	0.608
Pot diameter : Lines (P:L)	R	28	0.605	0.0216	0.865	0.663
Residual (R)	-	154	3.85	0.0250	-	-

The appropriate error term is given in each case. For simplicity, block and the 3-way interactions are not shown, although the 3-way interactions were never significant.

### Total mass of seeds

**Figure 4:**
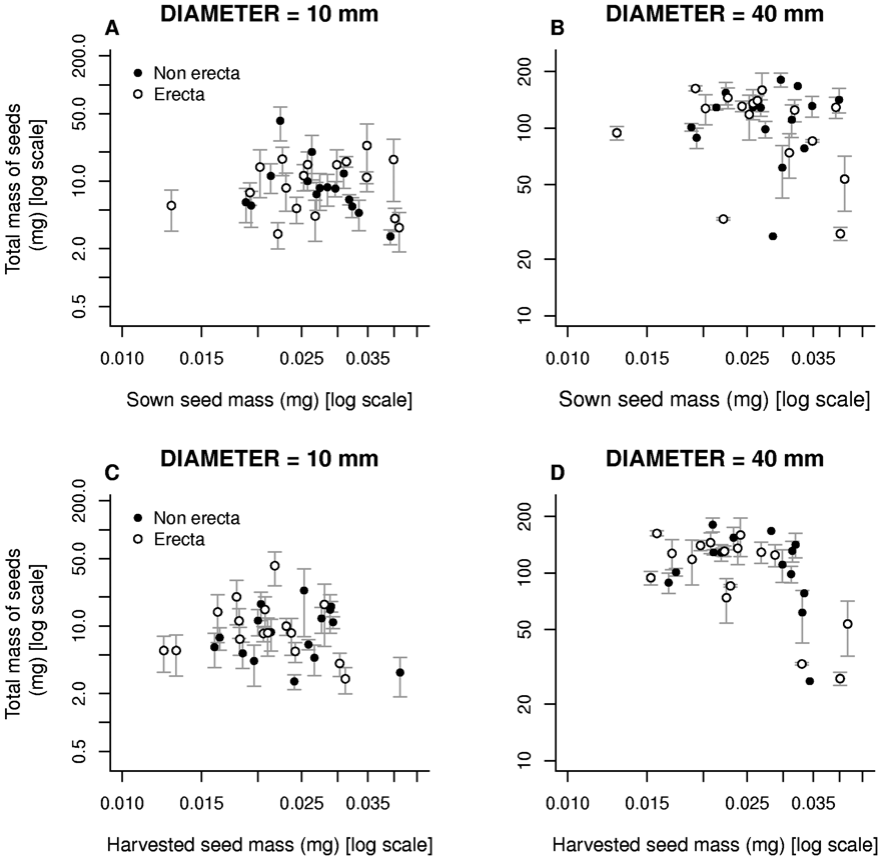
The relationship between the total mass of seeds produced and both sown seed mass and harvested seed mass. Points show the mean (±1 s.e.m) of all individuals belonging to the same line.

The relationship between sown seed mass and the total mass of seeds produced is shown in Figure 4. The total mass of seeds produced in 40-mm diameter pots was 14.4 (CI: 11.7–17.9) times larger than the total mass of seeds produced in 10-mm diameter pots, thus suggesting that adult size was primarily a function of pot size (the 40-mm diameter pot had a soil volume exactly 16-times greater than the 10-mm diameter pot). The total mass of seeds was unaffected by sown seed mass (Figure 4A–B), so that sown seed mass and adult mass are uncoupled (F_1,29_ = 1.55, *p* = 0.223, Table 4). The total mass of seeds was also not affected by the *erecta* mutation (F_1,29_ = 1.60, *p* = 0.216, Table 4) indicating no general fitness cost to this mutation. The pot size × lines interaction was effectively zero, but variation among lines was large (χ^2^ = 15.3). The relationship between harvested seed mass and the total mass of seeds is, however, different (Figure 4C–D). In this case there was a significant interaction between harvested seed mass and pot size (F_1,28_ = 7.80, *p* = 0.0093, Table 5): the slope of the relationship between harvested seed mass and the total mass of seeds produced was positive in 10-mm diameter pots (slope = 1.13±0.263), but flat in 40-mm diameter pots (slope = −0.17±0.319). Thus in very small pots, lines producing large seeds produced a greater total mass of seeds, while in larger pots, the total mass of seeds was independent of seed size. However, there was no relationship between the *sown* seed mass and the total mass of seeds in either pot size.

**Table 4:** ANOVA of the total mass of seeds with sown seed mass fitted as an explanatory variable.

Term	Error term	Df	Sum Squares	Mean Square	F	P
Pot diameter	R	1	376	376	681	<0.0001
*erecta* mutation	L	1	2.56	2.56	1.60	0.216
log (Sown seed mass)	L	1	2.48	2.48	1.55	0.223
Lines (L)	R	29	46.3	1.60	2.89	<0.0001
Pot diameter : *erecta* mutation	P:L	1	0.100	0.100	0.167	0.686
Pot diameter : log (Sown seed mass )	P:L	1	0.640	0.640	1.07	0.310
Pot diameter : Lines (P:L)	R	28	16.8	0.600	1.09	0.359
Residual (R)	-	154	85.1	0.550	-	-

The appropriate error term is given in each case. For simplicity, block and the 3-way interactions are not shown, although the 3-way interactions were never significant.

**Table 5:** ANOVA of the total mass of seeds with harvested seed mass fitted as an explanatory variable.

Term	Error term	Df	Sum Squares	Mean Square	F	P
Pot diameter	R	1	376.06	376.06	778	<0.0001
*erecta* mutation	L	1	2.56	2.56	1.27	0.269
log (Harvested seed mass)	L	1	0.72	0.72	0.356	0.555
Lines (L)	R	29	60.5	2.02	4.21	<0.0001
Pot diameter : *erecta* mutation	P:L	1	0.81	0.81	1.27	0.270
Pot diameter : log (Harvested seed mass )	P:L	1	4.99	4.99	7.8	0.00933
Pot diameter : Lines (P:L)	R	28	18.7	0.64	1.33	0.153
Residual (R)	-	154	45.5	0.48	-	-

The appropriate error term is given in each case. For simplicity, block and the 3-way interactions are not shown, although the 3-way interactions were never significant.

### Number of seeds

**Figure 5:**
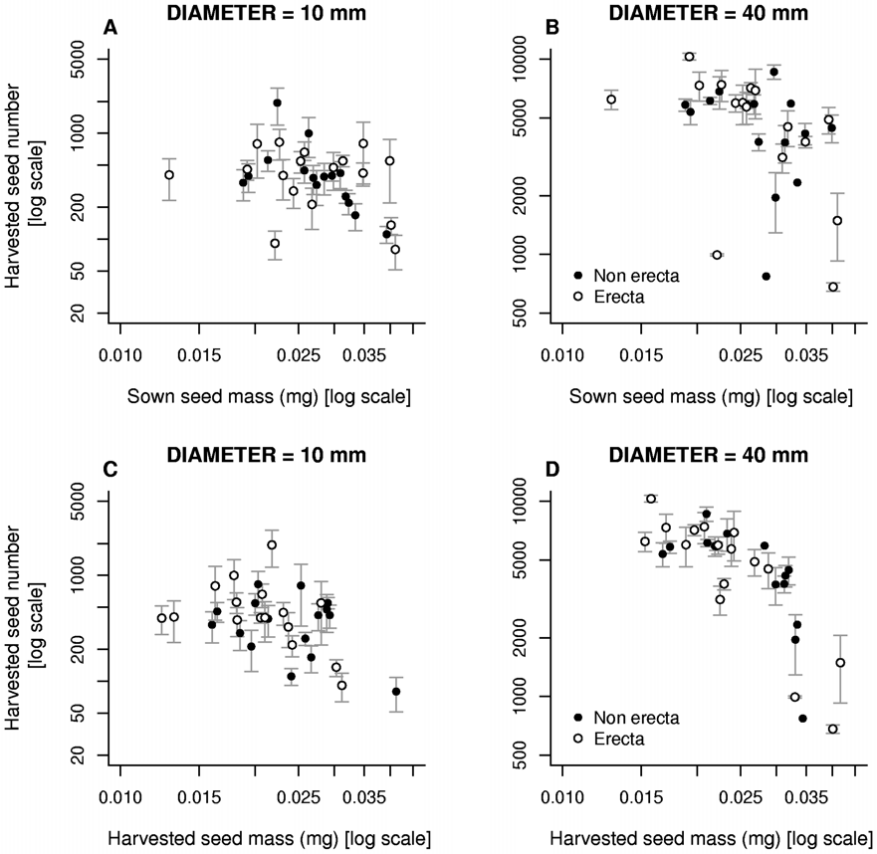
The relationship between the number of seeds produced and both sown seed mass and harvested seed mass. Points show the mean (±1 s.e.m) of all individuals belonging to the same line.

The slope of the relationship between sown seed mass and harvested seed number (Figure 5A–B) was very close to the expected value of −1 despite a large scatter (see eqn 2; slope: −1.02±0.775). Individuals produced 13.3 (CI: 10.7–16.1) times more seeds in 40-mm pots than in 10 mm pots again suggesting pot restriction. Carrying the *erecta* mutation did not affect the number of seeds a plant produced (F_1,29_ = 1.24, *p* = 0.0275, Table 6). The pot size × lines interaction was effectively zero, but again variation among lines was large (χ^2^ = 35.5). However, the relationships changed once harvested seed mass was fitted as the explanatory variable (Figure 5C–D). There was a significant interaction between harvested seed mass and pot size (F_1,28_ = 7.80, *p* = 0.0093, Table 7): the slope of the relationship between harvested seed mass and seed number was flat in 10-mm diameter pots (slope = 0.132±0.263) but negative in 40-mm diameter pots (slope = −1.166±0.319). Therefore lines producing large seeds have an advantage in small pots (they produce the same number of seeds, but these seeds are larger); however, the expected trade-off between seed size and number emerges in larger pots, with a slope close to the predicted value of −1.

**Table 6:** ANOVA of harvested seed number with sown seed mass fitted as the explanatory variable.

Term	Error term	Df	Sum Squares	Mean Square	F	P
Pot diameter	R	1	354.94	354.94	731	<0.0001
*erecta* mutation	L	1	2.71	2.71	1.24	0.275
log (Sown seed mass)	L	1	24.1	24.1	11.0	0.00244
Lines (L)	R	29	63.5	2.19	4.51	<0.0001
Pot diameter : *erecta* mutation	P:L	1	0.440	0.440	0.759	0.391
Pot diameter : log (Sown seed mass )	P:L	1	0.520	0.520	0.897	0.352
Pot diameter : Lines (P:L)	R	28	16.4	0.580	1.20	0.237
Residual (R)	-	154	74.8	0.490	-	-

The appropriate error term is given in each case. For simplicity, block and the 3-way interactions are not shown, although the 3-way interactions were never significant.

**Table 7:** ANOVA of harvested seed number with harvested seed mass fitted as the explanatory variable.

Term	Error term	Df	Sum Squares	Mean Square	F	P
Pot diameter	R	1	374.6	374.6	775	<0.0001
*erecta* mutation	L	1	2.53	2.53	1.25	0.272
log (Harvested seed mass)	L	1	9.54	9.54	4.72	0.0381
Lines (L)	R	29	60.5	2.02	4.21	<0.0001
Pot diameter : *erecta* mutation	P:L	1	0.98	0.98	2.65	0.115
Pot diameter : log (Harvested seed mass )	P:L	1	5.08	5.08	13.73	0.000920
Pot diameter : Lines (P:L)	R	28	10.6	0.37	0.758	0.801
Residual (R)	-	154	45.5	0.48	-	-

The appropriate error term is given in each case. For simplicity, block and the 3-way interactions are not shown, although the 3-way interactions were never significant.

## Discussion

The seed size/number trade-off is an important concept in life-history theory because it helps us to understand the astonishing variety of seed sizes found within and among plant communities [35], [36]. As laid out by Smith & Fretwell [1] once the resources available for reproduction are fixed the trade-off is inevitable: any increase in the size of individual offspring must be compensated for by a reduction in offspring number. However, if the trade-off is applied across genotypes or species producing different seed sizes, it could easily disappear if seed size is linked to adult size. This might explain why efforts to find the trade-off using multi-species data sets have often failed [3], [5].

The RIL population created by Alonso-Blanco et al. [23], [24] has the advantage of providing multiple genotypes from a single species that vary greatly in their seed mass due to genetic differences at around 11 different loci [23]; hence the trade-off between seed size and number within this population should be genetic. The RILs have the disadvantage of genetic correlations among traits; there are genetic correlations between seed size and vegetative traits such as rosette size, causing the trade-off between seed size and number to be steeper than expected from simple life-history theory (−1.68±0.18; *F_1,160_* = 84.4, *p*<0.0001; calculated from data in [23]). Thus, paradoxically, small-seeded genotypes produce more seeds in total because they delay flowering, produce longer leaves, build a larger rosette and hence accumulate more resources. However, *Arabidopsis* plants are normally raised under idealised conditions, in large pots with plentiful nutrients to allow full phenotypic expression of genetic characters: for example, in the Alonso-Blanco [23] study, plants were raised in clay pots of unknown depth filled with potting compost, and from the total mass of seeds reported, they clearly reached much higher final biomass than the plants we grew. Under such conditions, it seems reasonable to assume that much of the active growth phase is carbon limited rather than nutrient limited (and thus delaying flowering and continuing to increase rosette size leads to a greater total mass of seeds). However, under conditions of restricted space, a small rosette may easily provide enough carbon-fixation capacity to extract all the available belowground resources from the pot; delaying flowering and growing a larger rosette does not therefore lead to a greater mass of seeds. Thus, we suspected that the observed trade-off could be manipulated by changing the growth conditions. If plants are grown in poor soil, in a restricted space with no additional nutrients, then final size should be much more constrained by the availability of belowground resources. We reasoned that, under such conditions, small-seeded lines would not be able to accumulate more resources and hence final size would not vary with seed size across lines.

In line with our predictions, the final total mass of seeds was a simple multiple of pot size, and was unaffected by sown seed mass. This implies that, under such restrictive growth conditions, small-seeded lines do not apparently make larger rosettes and hence a greater mass of seeds. Likewise, under these conditions, the size advantage due to larger seeds does not persist into adulthood (otherwise plants from larger seeds would produce larger plants). This presumably occurs because large-seeded genotypes exhaust the resources of the pot more quickly, allowing smaller-seeded genotypes to catch up. This was nicely demonstrated by Susko and Cavers [10], who found that individuals from large seeds were larger after 15 days of growth in pots, but that such differences disappeared at later dates. Under such conditions, the only disadvantage to starting life from a smaller seed is the increased time to flowering and hence the associated increased risk of dying before reproduction [37]. In 10-mm diameter pots, there was no relationship between sown seed mass and the total mass of seeds, but there was a positive relationship between harvested seed mass and the total mass of seeds, although it is difficult to provide a plausible biological explanation for this result. This resulted in no relationship between seed size and number in the 10-mm diameter pots, and hence an advantage to large-seeded lines. In the larger 40-mm diameter pots, where the total mass of seeds was not related to seed size, we indeed found a relationship between harvested seed mass and harvested seed number (slope = −1.166±0.319) close to that expected from simple life-history theory (−1). The difference in the nature of the relationship that we uncovered, both between our two pot sizes and the experiment conducted by Alonso-Blanco et al. [23] seem to indicate that, as expected, the trade-off is highly sensitive to the environmental conditions.

However, just because large-seeded genotypes or species cannot maintain their size advantage when grown in pots, the situation can be very different in the field where large-seeded genotypes or species can maintain size differences over longer periods (e.g. [15], [38]). If such size differences could be maintained until reproduction, for example, through perfectly size-symmetric competition, then the final mass of plants could be directly proportional to their initial mass, potentially allowing seed mass to be a neutral trait [39,40,41,42]. Seed size could also potentially be a neutral trait if there is a perfect trade-off between the probability of survival and the number of seeds produced [43] although this requires the additional assumption that larger seeds never lead to larger plants [16]. Although these restrictions alone might make the idea of a neutral trade-off implausible, it is important to explore the necessary conditions for traits to be selectively neutral; the topical neutral theory of community ecology [40] requires not just that species are neutral, but that species traits are also neutral.

If seed size were a neutral trait and thus free to drift, this is a superficially attractive explanation for why similar species in the same environment have such a large variety of seed sizes [44], [45]. It might also explain why seed size/number trade-offs among individuals are sometimes difficult to detect in natural situations, because, with perfectly size-symmetric competition, the size hierarchy is maintained throughout the growing season. Thus, individuals from larger seeds make proportionally larger adult plants and the trade-off now appears per unit area of ground rather than per individual. However, if seed size were a neutral trait and free to drift among species it is difficult to understand the relative lack of plasticity within species; plants with additional resources tend to produce more seeds rather than larger ones [46], [47]. Similarly the variation in seed size within species [48] is dwarfed by the variation among species, implying that there is strong stabilising selection on seed size within species, as simple theory predicts [1], [6].

Currently, few good explanations exist for the variation in seed size found in *Arabidopsis thaliana* [23]. However, it is interesting to note that the original parent lines come from very different geographical locations, and that the main source of seed size variation in *Arabidopsis* is likely to be among, rather than within, different populations. For example, while it is true that the wildtype accessions are single individuals from their respective populations and hence may not be representative, it is tempting to speculate that the small-seeded Landsberg accession (from Northern Europe and from which the accession carrying the *erecta* mutation was derived) may be a product of a more urban environment, where suitable opportunities may often consist of cracks in pavements or gaps between cobble-stones. Here the amount of soil is limited and adult size is strongly constrained. In these circumstances, small-seeded species will tend to have higher fitness because the seed size/number trade-off operates among individuals, allowing small-seeded individuals to produce more seeds. In contrast, the large-seeded Cvi from tropical Africa is perhaps to be found in more stable environments with intense size-asymmetric competition; thus favouring larger seeds.

42. Turnbull LA, Rees M, Purves DW (2008) Why equalising trade-offs aren't always neutral. Ecology Letters 11: 1037-1046.

## Supporting Information

Table S1 Information about the 32 lines selected for the study.
(0.07 MB DOC)
