# Supplementary material for: The Effect of Growth Conditions on the Seed Size/Number Trade-Off
Source: PLoS One. 2009 Sep 10;4(9):e6917. doi: 10.1371/journal.pone.0006917 (PMC2735032; doi:10.1371/journal.pone.0006917)
Supplement: Table S1 — Information about the 32 lines selected for the study. (0.07 MB DOC) [file pone.0006917.s001.doc]

**Table S1.** Information about the 32 lines selected for the study. The two accessions L*er* and Cvi are the parents. The 30 remaining recombinant inbred lines are derived from reciprocal crosses between the two parents.

| NASC | RIL Koornneef | Published Seed Mass (*) [mg] | Sown Seed mass (**) [mg] | *ERECTA* mutation |
| --- | --- | --- | --- | --- |
| N8581 | L*er* | 0.0193 | 0.0202 | 1 |
| N8580 | Cvi | 0.0351 | 0.0348 | 0 |
| N22002 | CVL3 | 0.0162 | 0.0129 | 1 |
| N22014 | CVL15 | 0.0145 | 0.0193 | 0 |
| N22018 | CVL19 | 0.0251 | 0.0263 | 1 |
| N22026 | CVL27 | 0.0275 | 0.0270 | 1 |
| N22030 | CVL31 | 0.0295 | 0.0334 | 0 |
| N22033 | CVL34 | 0.0236 | 0.0297 | 0 |
| N22036 | CVL37 | 0.0325 | 0.0399 | 0 |
| N22037 | CVL38 | 0.0150 | 0.0188 | 0 |
| N22038 | CVL39 | 0.0202 | 0.0258 | 0 |
| N22043 | CVL44 | 0.0242 | 0.0285 | 0 |
| N22051 | CVL53 | 0.0327 | 0.0310 | 1 |
| N22057 | CVL60 | 0.0286 | 0.0393 | 1 |
| N22059 | CVL62 | 0.0190 | 0.0224 | 0 |
| N22094 | CVL124 | 0.0274 | 0.0252 | 1 |
| N22095 | CVL125 | 0.0200 | 0.0214 | 0 |
| N22098 | CVL128 | 0.0273 | 0.0274 | 0 |
| N22099 | CVL129 | 0.0243 | 0.0268 | 0 |
| N22105 | CVL135 | 0.0327 | 0.0348 | 1 |
| N22107 | CVL137 | 0.0302 | 0.0314 | 0 |
| N22109 | CVL139 | 0.0217 | 0.0231 | 0 |
| N22112 | CVL142 | 0.0315 | 0.0318 | 1 |
| N22124 | CVL154 | 0.0317 | 0.0323 | 0 |
| N22128 | CVL158 | 0.0373 | 0.0411 | 1 |
| N22130 | CVL160 | 0.0361 | 0.0402 | 1 |
| N22132 | CVL162 | 0.0256 | 0.0221 | 1 |
| N22138 | CVL168 | 0.0334 | 0.0299 | 0 |
| N22148 | CVL178 | 0.0207 | 0.0226 | 1 |
| N22149 | CVL179 | 0.0223 | 0.0243 | 1 |
| N22156 | CVL187 | 0.0183 | 0.0192 | 1 |
| N22160 | CVL191 | 0.0280 | 0.0257 | 1 |

(*) Source : Alonso-Blanco C, Blankestijn-De Vries H, Hanhart CJ, Koornneef M (1999) Natural allelic variation at seed size loci in relation to other life history traits of *Arabidopsis thaliana*. Proceedings of the National Academy of Sciences of the United States of America 96: 4710-4717.

(**) Source: The Arabidopsis Information Resource (TAIR).
